# miR-205a mediated suppression of CDH11 disrupts Wnt/β-catenin signaling and impairs chondrocyte differentiation

**DOI:** 10.1038/s41420-026-03146-3

**Published:** 2026-05-06

**Authors:** Kai Liu, Buyun Chen, Junhong Hou, Yuanliang Li, Lihong Ning, Shaochuan Li, Ying Li, Aoyun Li, Quazi T. H. Shubhra, Hui Zhang

**Affiliations:** 1 College of Veterinary Medicine, South China Agricultural University, Guangzhou, China; 2 Xizang Animal Disease Prevention and Control Center, Lasa, China; 3 College of Veterinary Medicine, Henan Agricultural University, Zhengzhou, China; 4 Institute of Chemistry, University of Silesia in Katowice, Katowice, Poland; 5 College of Animal Science, Xizang Agricultural and Animal Husbandry University, Linzhi, China

**Keywords:** Developmental biology, Molecular biology

## Abstract

The precise regulation of chondrocyte differentiation is critical for endochondral ossification, and its disruption underlies a spectrum of skeletal diseases. While the Wnt/β-catenin signaling pathway is a well-established master regulator of skeletal development, its precise regulation during chondrogenesis remains incompletely understood. Here, we identify a novel regulatory axis centered on microRNA-205a and its target, the adhesion molecule Cadherin-11 (CDH11), in avian embryonic models. We demonstrate that CDH11 functions as a positive regulator of chondrocyte differentiation by promoting Wnt/β-catenin signaling. Conversely, miR-205a acts as a potent endogenous inhibitor of this process. Through dual-luciferase reporter assays, we confirm that miR-205a directly binds to the 3’UTR of CDH11 mRNA. Functional studies revealed that miR-205a overexpression suppresses chondrogenesis by downregulating CDH11, thereby inhibiting the Wnt/β-catenin pathway and key chondrogenic markers like Runx2 and BMP2. Silencing miR-205a or overexpressing CDH11 produced the opposite effect, promoting the differentiation program. Critically, rescue experiments using a Wnt/β-catenin pathway agonist substantiated that miR-205a exerts its inhibitory effects primarily through modulating this pathway. Our findings delineate a conserved miR-205a/CDH11/Wnt-β-catenin regulatory circuit that is essential for chondrocyte differentiation, offering fundamental new insights into the molecular etiology of cartilage development and its associated disorders.

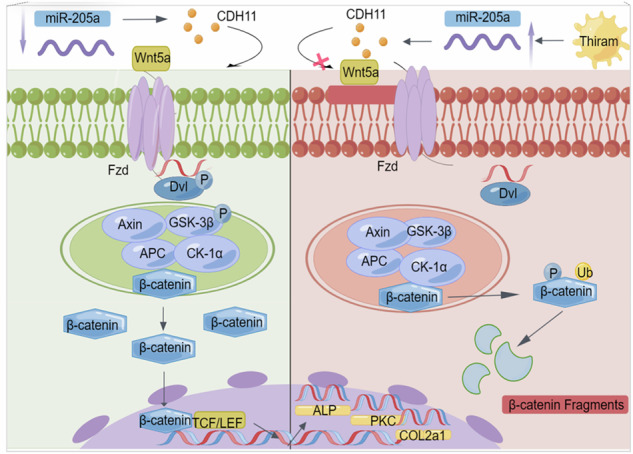

## Introduction

As a vital component of the vertebrate musculoskeletal system, the structural stability of bones relies on highly organized connective tissue. The cartilaginous layer comprises diverse chondrocytes and extracellular matrix, while bone tissue forms a rigid honeycomb structure through mineral deposition. Together, these elements maintain the mechanical function and metabolic homeostasis of the skeleton [1]. During the early embryonic development of poultry (such as broiler chickens), the tibia exists as a cartilaginous template primarily composed of chondrocytes and the matrix they secrete [2]. As the growth plate undergoes rapid proliferation, this cartilaginous framework gradually establishes itself, laying the foundation for subsequent ossification [3]. Following hatching, the process of endochondral ossification commences. Chondrocytes undergo proliferation and differentiate toward hypertrophy, simultaneously synthesizing and secreting large quantities of key components such as type II collagen (Col2A1) and aggrecan, thereby forming a functional chondrocyte extracellular matrix (ECM) [4, 5]. During this process, blood vessels gradually invade the cartilage template, transporting osteoblasts derived from bone marrow mesenchymal stem cells and osteoclasts formed by monocyte fusion into the cartilage interior, thereby promoting cartilage vascularization [6, 7]. Ultimately, as blood vessels and osteoblasts invade the hypertrophic cartilage region, chondrocytes in this area undergo apoptosis, initiating the process of matrix mineralization. This gradually replaces cartilage tissue with bone tissue, enabling the longitudinal growth of bones. This series of biological events is precisely regulated by the intricate coordination of multiple signaling pathways, including the PTHrP-Ihh axis, the Wnt/β-catenin pathway, and the BMP signaling pathway. Together, these pathways maintain the homeostasis of skeletal development by regulating the proliferation, differentiation, maturation, and mineralization balance of chondrocytes [8–10]. However, as a common skeletal developmental disorder, achondroplasia is characterized by the failure of chondrocytes in the growth plate to undergo successful hypertrophic differentiation [11]. This leads to the abnormal accumulation of immature chondrocytes, which impedes the normal vascular invasion and mineralization processes within the cartilage matrix. Ultimately, this affects both linear bone growth and structural integrity [12].

MicroRNAs (miRNAs) are endogenous non-coding short RNA molecules that regulate gene expression at the post-transcriptional level by specifically binding to their target mRNAs. They play crucial roles in various biological processes and demonstrate significant potential as disease biomarkers and therapeutic targets [13–15]. Among these, miR-205 is widely recognized as a tumor suppressor, exhibiting abnormal expression in various malignant tumors. For instance, overexpression of miR-205 in breast cancer significantly inhibits cell proliferation and induces apoptosis. Similarly, its tumor-suppressing function has also been observed in renal cell carcinoma and melanoma [16, 17]. Notably, miR-205a exhibits a high expression pattern in chicken embryos, suggesting it may play a specific regulatory role during embryonic development [18]. On the other hand, cadherin 11 (CDH11), as a member of the cadherin superfamily, is extensively involved in regulating various biological processes. For instance, it exhibits dual roles in multiple tumor types and influences the development and function of the nervous system [19, 20]. Additionally, CDH11 plays a crucial role in the skeletal system, performing key functions in maintaining bone homeostasis and regulating osteoblast differentiation [21]. The WNT signaling cascade serves as a primary regulator of development across the entire animal kingdom and is implicated in the pathogenesis of various growth-related pathologies and cancers [22, 23]. The Wnt/β-catenin signaling pathway is a key pathway known to be involved in osteogenic differentiation and bone anabolic metabolism, and its abnormal activation is closely associated with various skeletal disorders [24, 25].

Currently, there is limited research on the regulatory role and molecular mechanisms of miR-205a in cartilage development, particularly its function in avian embryonic models remains poorly understood. Furthermore, miR-205, as a tumor suppressor, its effect on tibial dyschondroplasia (TD) is still largely speculative, with no in-depth studies conducted to date. Therefore, this study aims to investigate whether miR-205a regulates the differentiation process of chicken embryonic chondrocytes by inhibiting the Wnt/β-catenin signaling pathway through targeting CDH11. This study not only helps reveal the novel function and regulatory network of miR-205a in chondrogenesis, but also provides a theoretical basis for understanding the molecular mechanisms underlying skeletal development and related diseases. Furthermore, it may offer potential targets and strategies for skeletal regenerative medicine and cartilage injury repair.

## Results

### A tibial chondrodysplasia model reveals dysregulation of the Wnt/β-catenin pathway and impaired cartilage differentiation

**Fig. 1:**
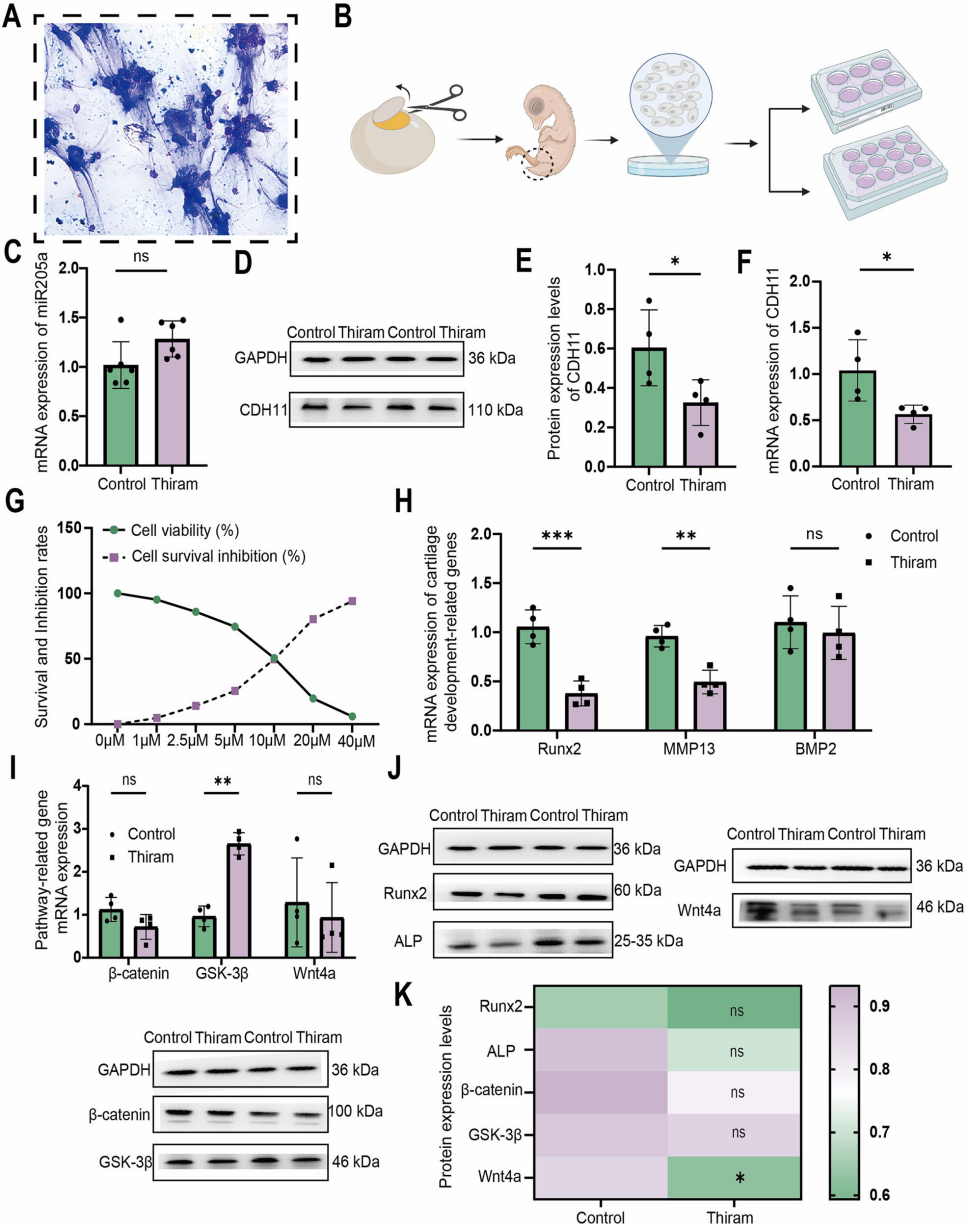
Dysregulation of the Wnt/β-catenin pathway accompanied by impaired cartilage differentiation in a tibial chondrodysplasia model. **A** Toluidine blue staining reveals typical chondrocyte morphology in vitro cultured cells. **B** Schematic diagram of chicken embryo tibial chondrocyte isolation and primary culture. **C** miR-205a expression is upregulated in chondrodysplasia models (*n* = 6). **D**, **E** Western blot analysis demonstrates reduced CDH11 protein expression in the model group (**D**, representative blot. **E** quantitative results, *n* = 4). **F** qPCR confirmed reduced CDH11 mRNA expression (*n* = 4). **G** CCK-8 assay indicates toxic effects of thiram on chondrocyte viability (*n* = 6); **H**, **I** qPCR analysis revealed abnormal expression of chondrogenic differentiation marker genes (**H**, *n* = 4) and Wnt/β-catenin pathway-related genes (**I**, *n* = 4). **J**, **K** Western blot detection of key proteins involved in chondrogenic differentiation and the Wnt pathway (**J**, representative blot. **K**, quantitative analysis, *n* = 3). Experiments were repeated three times. Each dot on the chart represents a data point. Data were analyzed with unpaired t-test (**C**, **E**, **F**, **H**, **I**, **K**) and represented with mean ± SEM unless indicated. *P < 0.05, **P < 0.01, ***P < 0.001, ****P < 0.0001.

To elucidate the dysregulation of the Wnt/β-catenin signaling pathway in a TD model and its impact on chondrogenic differentiation, we first established an in vitro thiram-induced chondrocyte model. Chondrocyte identity was confirmed by toluidine blue staining, which revealed characteristic cellular morphology and surrounding extracellular matrix (Fig. 1A). We also established the chondrocyte isolation protocol (Fig. 1B). qRT-PCR analysis revealed an upward trend in miR-205a expression in primary chondrocytes treated with thiram, though this increase did not reach statistical significance (Fig. 1C). Subsequently, to further investigate the expression changes of CDH11 in the TD model, we conducted analyses at both the transcriptional and protein levels. Western blot results demonstrated a significant reduction in CDH11 protein expression (Fig. 1D, E), while its mRNA levels showed a decrease (Fig. 1F). In the TD model, thiram showed dose-dependent effects on chondrocyte viability, with reduced viability at higher concentrations (Fig. 1G). Furthermore, qRT-PCR results showed that the expression levels of *Runx2* and *MMP13* were significantly downregulated in the TD group, while *BMP2* expression was downregulated but not significantly (Fig. 1H). mRNA expression of pathway-related genes *Wnt4a* and *β-catenin* was downregulated, while *GSK-3β* mRNA levels were significantly elevated (Fig. 1I). At the protein level, Western blot analysis further confirmed a significant reduction in Wnt4a expression. Although GSK-3β protein showed an upward trend, it did not reach statistical significance. Chondrogenic-related proteins Runx2 and ALP exhibited a downward trend but remained non-significant (Fig. 1J–K). In summary, these results indicate that thiram treatment promotes chondrocyte proliferation while simultaneously inhibiting their differentiation process.

### miR-205a is a key regulator of chondrocyte differentiation, with gain- and loss-of-function studies confirming its suppression of the Wnt/β-catenin pathway

**Fig. 2:**
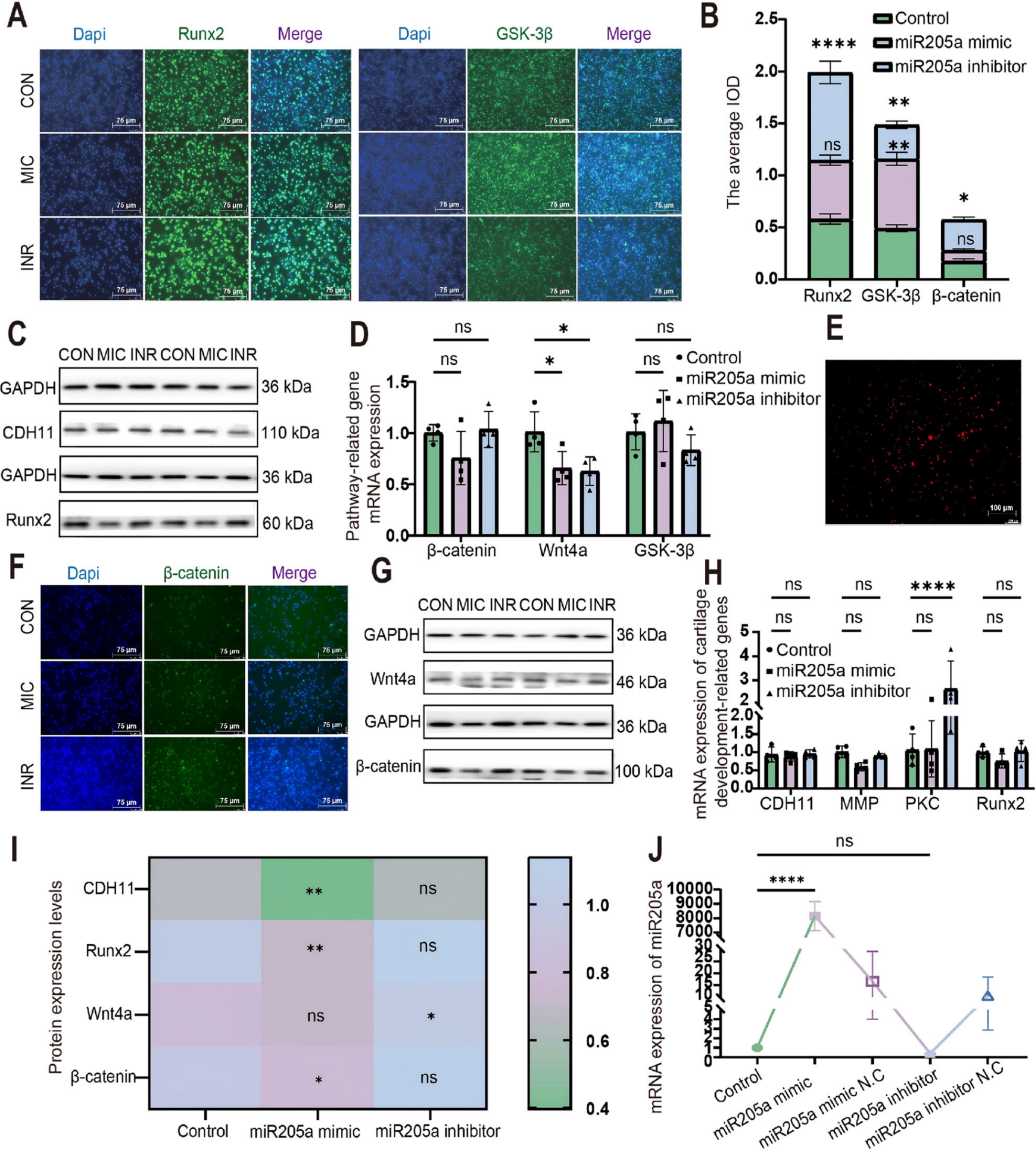
miR-205a influences chicken embryonic chondrocyte differentiation by regulating key factors in the Wnt/β-catenin pathway. **A**, **B** Immunofluorescence staining reveals the effects of miR-205a mimic and inhibitor treatments on the subcellular localization (**A**, scale bar: 75 μm) and expression levels (**B**, *n* = 3) of cartilage differentiation markers and proteins associated with the Wnt/β-catenin pathway. **C** Western blot analysis of miR-205a-regulated expression changes in chondrogenic differentiation-related proteins. **D** qRT-PCR detection of mRNA expression levels in Wnt/β-catenin pathway-related genes under miR-205a gain-of-function and loss-of-function conditions (*n* = 4). **E** Fluorescence microscopy validation of miR-205a inhibitor transfection efficiency. **F** Immunofluorescence showing effects of miR-205a mimic and inhibitor on GSK-3β protein localization (scale bar: 75 μm). **G** Western blot analysis of expression changes in key Wnt/β-catenin signaling proteins after miR-205a treatment. **H** qRT-PCR detection of miR-205a mimic and inhibitor effects on chondrogenic differentiation-related gene expression (*n* = 4). **I** Statistical analysis showing relative expression levels of chondrogenic differentiation and pathway-related proteins across groups (*n* = 3). **J** qRT-PCR validation of miR-205a mimic and inhibitor transfection efficiency. Experiments were repeated three times. Each dot on the chart represents a data point. Data were analyzed with one-way ANOVA was conducted, followed by Dunnett’s t-tests for multiple comparisons (**B**, **D**, **H**–**J**) and represented with mean ± SEM unless indicated. *P < 0.05, **P < 0.01, ***P < 0.001, ****P < 0.0001.

To investigate the function of miR-205a in chondrocytes, we successfully established chondrocyte models overexpressing and under expressing miR-205a by transfecting miR-205a mimics and CY3-labeled miR-205a inhibitors, respectively. Under fluorescence microscopy, distinct red (CY3) signals were observed, confirming transfection efficiency (Fig. 2E). Immunofluorescence staining revealed that miR-205a overexpression significantly reduced the fluorescence signals of Runx2 and β-catenin proteins while markedly increasing GSK-3β expression; conversely, miR-205a inhibition markedly enhanced Runx2 and β-catenin signaling and reduced GSK-3β expression (Fig. 2A, B, F). Western blot analysis revealed that in chondrocytes treated with the miR-205a mimic, β-catenin protein levels were significantly downregulated, while Wnt4a protein levels were also downregulated but not significantly. Following miR-205a silencing, Wnt4a protein levels were significantly upregulated, and β-catenin protein levels were also increased, though this increase did not reach statistical significance. Furthermore, following miR-205a overexpression, both CDH11 and Runx2 protein expression were significantly downregulated (Fig. 2C, G, I). At the gene expression level, qPCR analysis revealed that miR-205a mimic treatment significantly reduced *Wnt4a* mRNA levels, showed a trend toward downregulation of *β-catenin*, and upregulated *GSK-3β*, though the latter change did not reach statistical significance. In contrast, the inhibitor treatment group exhibited opposite trends in these gene expressions (Fig. 2D). Concurrently, miR-205a mimic treatment reduced mRNA levels of *CDH11*, *MMP*, *Runx2*, and *PKC*. In the inhibitor-treated group, *PKC* expression was significantly upregulated, while *CDH11*, *MMP*, and *Runx2* expression showed an upward trend but remained non-significant (Fig. 2H). qPCR results validated that miR-205a mimic expression was significantly upregulated, whereas the inhibitor exhibited a downward trend. (Fig. 2J).

### miR-205a inhibitors reverse chondrodysplasia and restore abnormal Wnt/β-catenin signaling

**Fig. 3:**
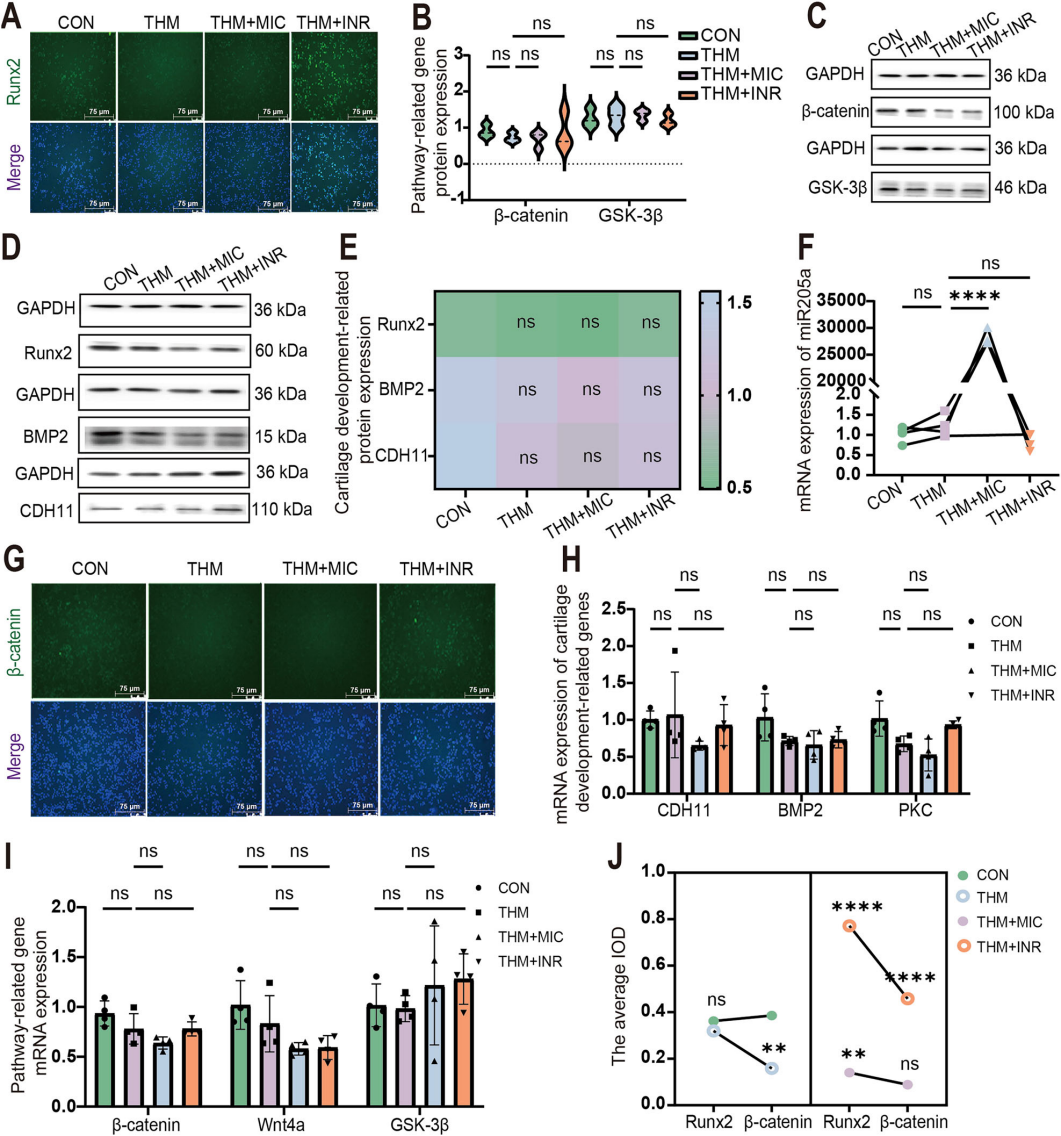
miR-205a inhibitor rescues thiram-induced cartilage differentiation defects and reverses Wnt/β-catenin signaling pathway abnormalities. **A** Immunofluorescence staining reveals effects of miR-205a mimic and inhibitor on expression and localization of chondrogenic marker proteins in thiram-induced dysplasia models (scale bar: 75 μm); **B**, **C** Western blot analysis of Wnt/β-catenin pathway-related protein expression changes (**B**, *n* = 3) and representative blot (**C**); **D**, **E** Western blot analysis of chondrogenic differentiation-related protein expression (**D**) and quantitative results (**E**, *n* = 3); **F** qPCR detection of miR-205a mimic and inhibitor effects on their own expression levels in the dysplasia model (*n* = 4). **G** Immunofluorescence staining showing subcellular localization changes of β-catenin in the miR-205a-regulated group (scale bar: 75 μm); **H** qPCR analysis of mRNA expression of chondrogenic differentiation-related genes in thiram model and miR-205a-regulated groups (*n* = 4). **I** qPCR detection of expression levels of downstream genes in the Wnt/β-catenin pathway across corresponding treatment groups (*n* = 4). **J** Immunofluorescence quantitative analysis revealing expression trends of key proteins (*n* = 3). Experiments were repeated three times. Each dot on the chart represents a data point. Data were analyzed with one-way ANOVA was conducted, followed by Dunnett’s t-tests for multiple comparisons (**B**, **E**, **F**, **H**–**J**) and represented with mean ± SEM unless indicated. *P < 0.05, **P < 0.01, ***P < 0.001, ****P < 0.0001.

To investigate the regulatory mechanisms of thiram and miR-205a in chondrogenesis, we treated chicken embryonic chondrocytes with 5 μM thiram and co-transfected them with either miR-205a mimic or miR-205a inhibitor to achieve simultaneous overexpression and suppression of miR-205a under thiram-induced conditions. Immunofluorescence staining results revealed that compared to the thiram group, co-treatment with thiram and miR-205a mimic significantly reduced the protein expression level of Runx2, whereas co-treatment with thiram and miR-205a inhibitor significantly increased it. Similarly, β-catenin fluorescence signals were attenuated in the mimic co-treatment group but significantly enhanced in the inhibitor co-treatment group (Fig. 3A, G, J). Western blot results showed that miR-205a overexpression downregulated β-catenin protein expression, while GSK-3β exhibited an upward trend that did not reach statistical significance. Conversely, miR-205a inhibition upregulated β-catenin protein expression, with GSK-3β showing an opposite trend that also failed to reach statistical significance (Fig. 3B, C). Results related to cartilage development proteins showed that miR-205a overexpression downregulated the protein expression of Runx2 and BMP2, while miR-205a inhibition exhibited an opposite trend, though not statistically significant. Notably, the protein levels of CDH11 showed a decreasing trend in the mimic group and an increasing trend in the inhibition group, but the changes were not significant (Fig. 3D, E). qRT-PCR analysis revealed that co-transfection of miR-205a mimic with thiram dramatically increased miR-205a expression by approximately 25,000-fold (Fig. 3F). At the mRNA level, co-treatment with thiram and miR-205a mimic led to decrease expression of *BMP2* and *PKC*, whereas inhibitor treatment showed the opposite trend. Transcriptional levels of *CDH11* were also downregulated in the mimic group and upregulated in the inhibitor group (Fig. 3H). Concurrently, expression of *β-catenin* and *Wnt4a* decreased in the mimic group, while *GSK-3β* expression was upregulated; inhibitor treatment reversed this effect (Fig. 3I). In summary, these findings suggest that miR-205a inhibition significantly alleviates thiram-induced chondrodysplasia and promotes activation of the Wnt/β-catenin signaling pathway, whereas its overexpression produces the opposite effect. This further confirms that miR-205a plays a crucial role in this pathway by targeting CDH11.

### The Wnt/β-catenin pathway activator LiCl suppresses disease phenotypes and antagonizes the anti-differentiation function of miR-205a

**Fig. 4:**
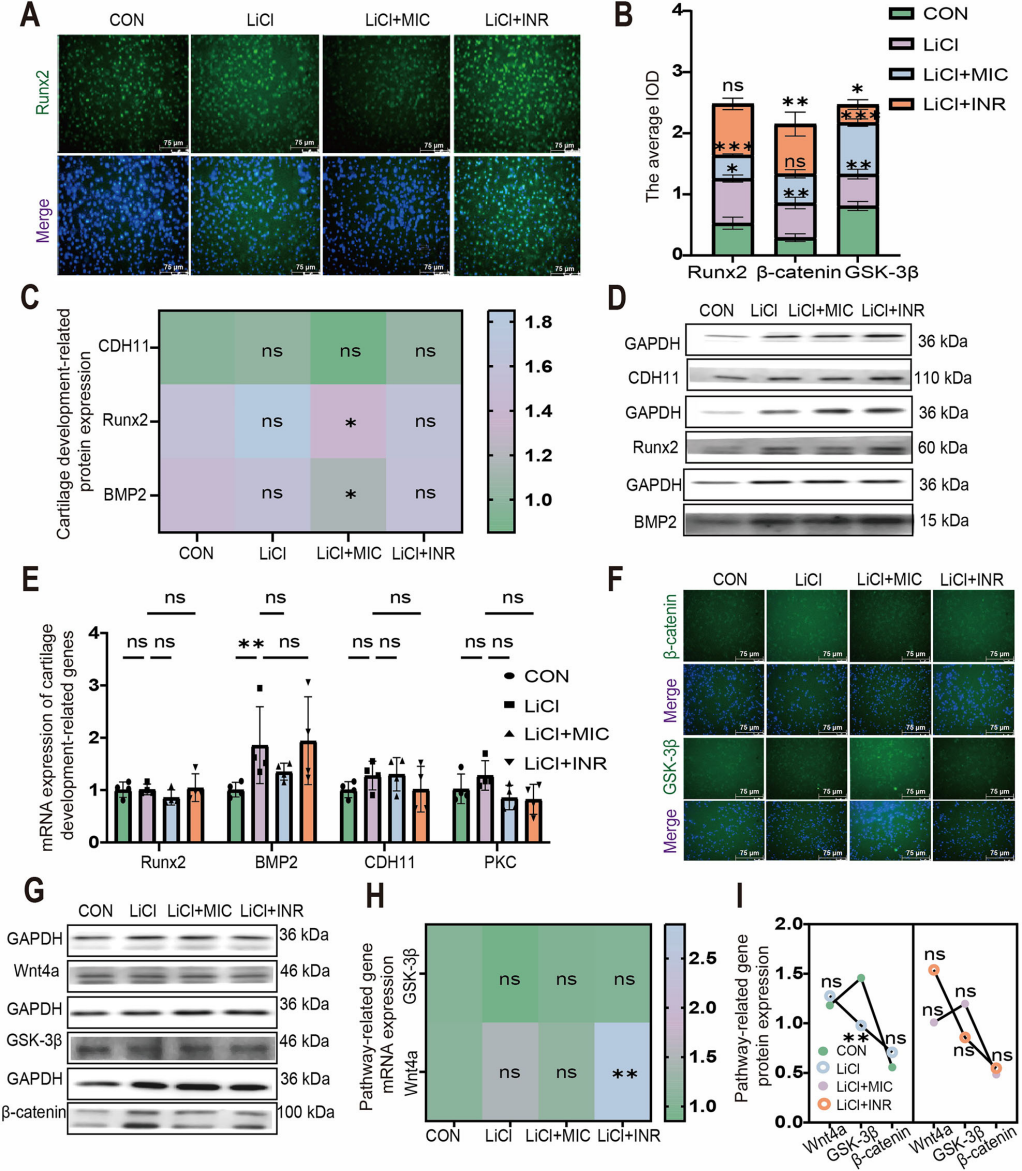
The Wnt/β-catenin pathway activator LiCl reverses the regulatory effect of miR-205a on the differentiation of chicken embryonic chondrocytes. **A** Immunofluorescence staining showing cellular localization of chondrogenic differentiation-related proteins after co-treatment with LiCl and miR-205 mimic or inhibitor (scale bar: 75 μm). **B** Quantitative analysis of corresponding protein immunofluorescence intensity (*n* = 3); **C**, **D** Western blot detection of chondrogenic differentiation-related protein expression levels (**C**, *n* = 3) and representative blot (**D**). **E** qPCR analysis of mRNA expression changes in chondrogenic differentiation-related genes across groups (*n* = 4). **F** Immunofluorescence staining showing subcellular localization of key Wnt/β-catenin pathway proteins after co-treatment with LiCl and miR-205 mimic/inhibitor (scale bar: 70 μm). **G** Representative Western blot showing expression of pathway-related proteins. **H** qPCR detection of transcription levels of Wnt/β-catenin pathway downstream genes (*n* = 4). **I** Western blot quantitative analysis of pathway-related protein expression levels (*n* = 3). Experiments were repeated three times. Each dot on the chart represents a data point. Data were analyzed with one-way ANOVA was conducted, followed by Dunnett’s t-tests for multiple comparisons (**B**, **C**, **E**, **H**–**I**) and represented with mean ± SEM unless indicated. *P < 0.05, **P < 0.01, ***P < 0.001, ****P < 0.0001.

To elucidate the regulatory effects of LiCl on the Wnt/β-catenin signaling pathway and genes associated with chondrocyte differentiation, we treated cells with 10 mM LiCl and examined its effects in combination with either a miR-205a mimic or inhibitor. Immunofluorescence staining confirmed that LiCl treatment significantly increased the positive signal rates of Runx2 and β-catenin while markedly suppressing GSK-3β expression. In the miR-205a inhibitor co-treatment group, Runx2 and β-catenin expression were further upregulated, whereas GSK-3β expression was more strongly suppressed (Fig. 4A, B, F). Western blot results showed that LiCl treatment upregulated the protein expression of CDH11, Runx2, and BMP2, with this effect being more pronounced in the miR-205a inhibitor group (Fig. 4C, D). qRT-PCR analysis revealed that mRNA expression of *Runx2*, *CDH11*, and *PKC* showed an upward trend in the LiCl-treated group alone, though not statistically significant, while *BMP2* mRNA expression was significantly upregulated. In contrast, co-treatment with LiCl and miR-205a mimic resulted in a slight downregulation of mRNA expression for these genes, which was not statistically significant (Fig. 4E). Concurrently, pathway-associated protein results revealed that LiCl treatment upregulated Wnt4a and β-catenin protein expression, with a more pronounced effect in the miR-205a inhibitor group. Conversely, GSK-3β showed significant downregulation at the protein level in the LiCl group and upregulation in the miR-205a mimic group (Fig. 4G–I). The pathway-associated gene *GSK-3β* showed a downward trend in mRNA expression in the LiCl group, while *Wnt4a* exhibited an upward trend, though neither reached statistical significance. In the miR-205a inhibitor group, *Wnt4a* was significantly upregulated (Fig. 4H).

### miR-205a mediates its regulation of the Wnt/β-catenin pathway and cartilage differentiation by directly targeting CDH11

**Fig. 5:**
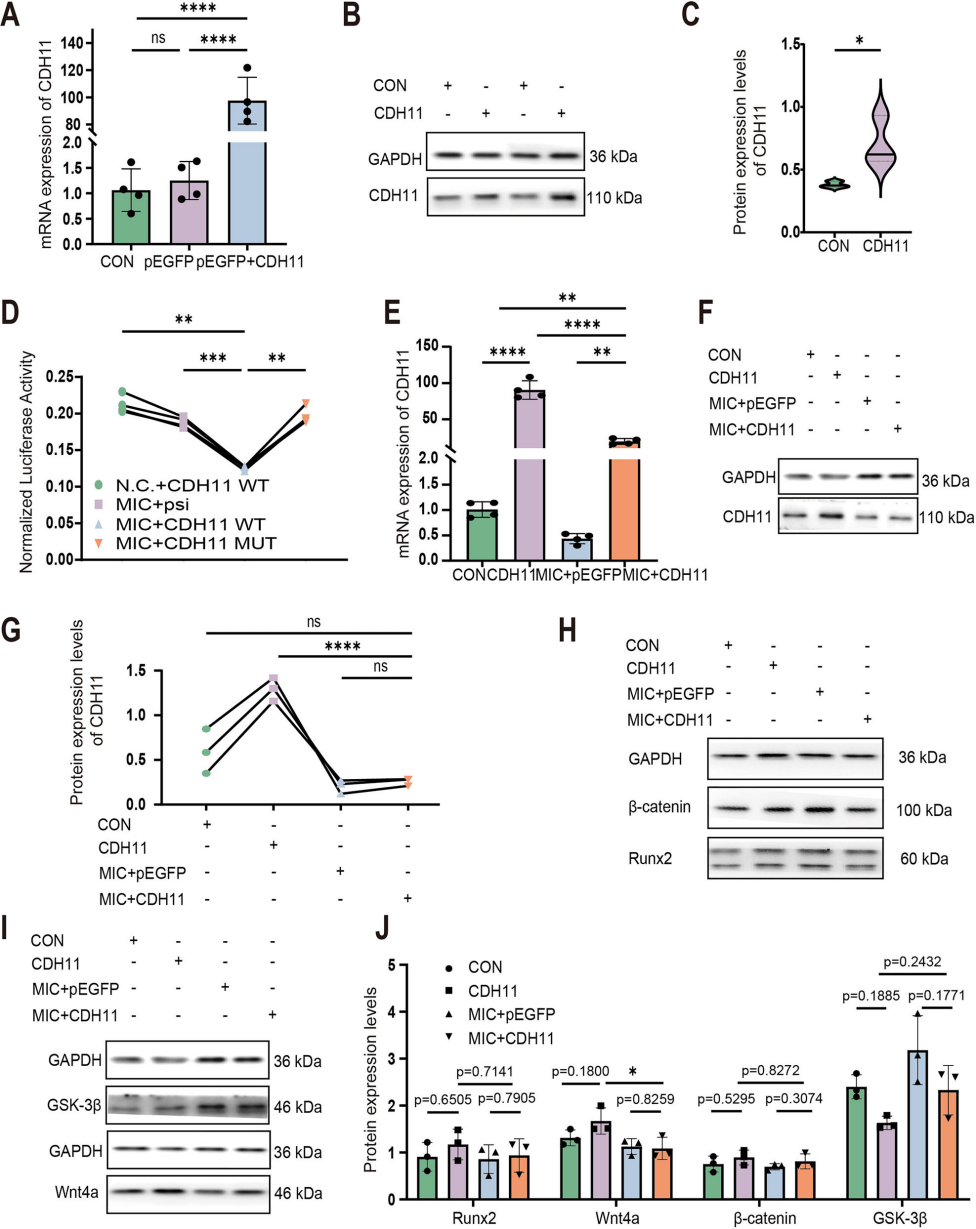
miR-205a inhibits the Wnt/β-catenin pathway and promotes chondrogenic differentiation by directly targeting CDH11. **A** qPCR detection of CDH11 mRNA expression levels after overexpression (*n* = 4). **B**, **C** Western blot analysis showing CDH11 protein band (**B**) and expression levels (**C**, *n* = 3) after overexpression; (**D**) dual luciferase reporter assay validating CDH11 as a direct target gene of miR-205a. **E** qPCR analysis of CDH11 mRNA expression after co-transfection with miR-205a mimic and CDH11 overexpression vector (*n* = 4); **F**, **G** CDH11 protein band (**F**) and expression level changes (**G**, n = 3) after co-transfection of CDH11 overexpression and miR-205a mimic. **H**–**J** co-transfection of CDH11 overexpression vector and miR-205a mimic showed cartilage differentiation and Wnt/β-catenin pathway-related protein bands (**H**, **I**) and quantitative analysis (**J**, *n* = 3); Experiments were repeated three times. Each dot on the chart represents a data point. Data were analyzed with one-way ANOVA was conducted, followed by Dunnett’s t-tests for multiple comparisons (**A**, **C**–**E**, **J**) and represented with mean ± SEM unless indicated. *P < 0.05, **P < 0.01, ***P < 0.001, ****P < 0.0001.

To elucidate the role of CDH11 in regulating Wnt/β-catenin signaling and the expression of genes associated with chondrocyte differentiation, we constructed a CDH11 overexpression vector and co-transfected it with a miR-205a mimic into chicken embryonic chondrocytes. The mRNA level confirmed that transfection with the CDH11 overexpression vector alone significantly increased CDH11 transcription (Fig. 5A). At the Western blot analysis level, transfection with the CDH11 overexpression vector alone significantly increased CDH11 protein expression levels (Fig. 5B, C). Dual luciferase reporter assay results indicate that miR-205a specifically binds to the wild-type 3’ UTR sequence of CDH11 and significantly inhibits its luciferase activity (Fig. 5D). Co-transfection of the CDH11 overexpression vector with the miR-205a mimic markedly reduced CDH11 mRNA expression (Fig. 5E). Co-transfection of the CDH11 overexpression vector with the miR-205a mimic significantly reduced CDH11 protein expression levels (Fig. 5F, G). Additionally, in terms of protein expression, the co-transfection group of CDH11 overexpression and miR-205a showed lower levels of Runx2 and β-catenin compared to the CDH11 overexpression alone group, but higher levels than the miR-205a overexpression alone group. The differences between groups were not significant, whereas GSK-3β exhibited the opposite trend. Meanwhile, Wnt4a protein expression was significantly lower in the co-transfection group compared to the CDH11 overexpression group, while showing an upward trend relative to the miR-205a overexpression group (Fig. 5H–J).

**Fig. 6:**
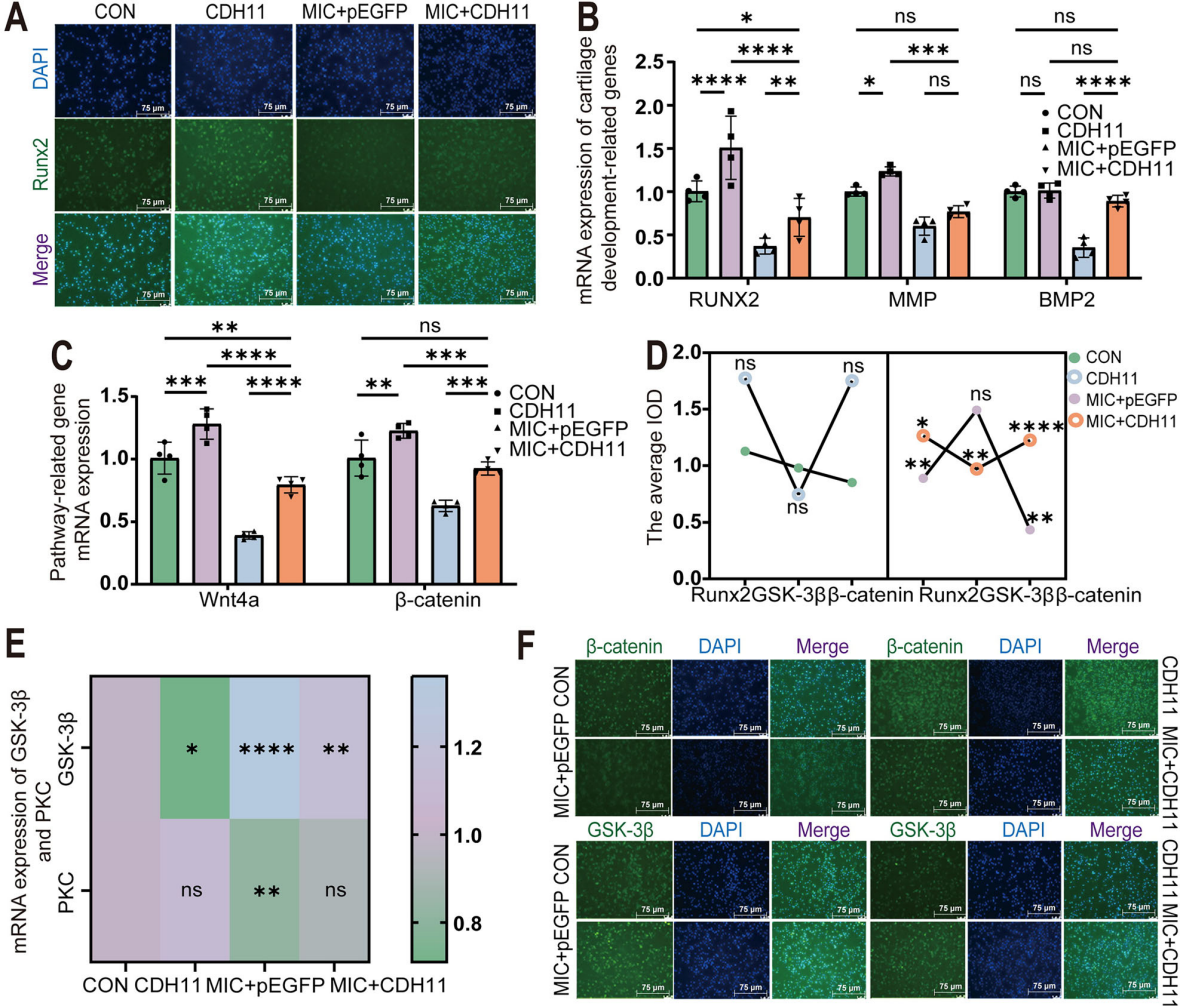
miR-205a regulates the Wnt/β-catenin pathway and chondrocyte differentiation by targeting CDH11. **A** Immunofluorescence staining reveals the cellular localization of chondrogenic proteins following co-treatment with CDH11 overexpression and miR-205 mimic (scale bar: 75 μm). **B** qPCR analysis of mRNA expression levels for cartilage differentiation-related genes in each group (*n* = 4). **C** qPCR detection of mRNA expression changes for Wnt/β-catenin pathway-related genes in each group (*n* = 4). **D** Immunofluorescence quantitative analysis showing key protein expression levels (*n* = 3). **E** qPCR analysis of mRNA expression of GSK-3β and PKC genes in each group (*n* = 4). **F** Immunofluorescence staining showing cellular localization of Wnt/β-catenin pathway-related proteins after co-treatment with CDH11 overexpression and miR-205 mimic (scale bar: 75 μm). Experiments were repeated three times. Each dot on the chart represents a data point. Data were analyzed with one-way ANOVA was conducted, followed by Dunnett’s t-tests for multiple comparisons (**B**–**E**) and represented with mean ± SEM unless indicated. *P < 0.05, **P < 0.01, ***P < 0.001, ****P < 0.0001.

Continuing to explore the CDH11 on the Wnt signaling pathway and chondrocyte-specific gene expression, we performed qRT-PCR and immunofluorescence assays. Immunofluorescence analysis of Runx2 revealed that CDH11 overexpression significantly increased its protein expression levels. In contrast, co-transfection of CDH11 with a miR-205a mimic markedly attenuated this effect (compared to the CDH11 overexpression group), yet expression levels remained significantly higher than in the miR-205a mimic alone group (Fig. 6A, D). qRT-PCR results revealed that CDH11 overexpression significantly promoted mRNA expression of *Runx2* and *MMP*. In contrast, the co-treatment group with CDH11 overexpression and miR-205a mimic showed significantly downregulated transcription levels of *Runx2* and *MMP*, while the transcription level of *BMP2* decreased but not significantly (Fig. 6B). Furthermore, overexpression of CDH11 significantly upregulated the mRNA expression of *Wnt4a* and *β-catenin*, whereas co-treatment with CDH11 overexpression and miR-205a mimetic markedly downregulated their mRNA expression (Fig. 6C). Concurrently, CDH11 overexpression increased *PKC* mRNA expression, whereas co-treatment with CDH11 overexpression and miR-205a mimic reduced its mRNA expression. Under both intervention conditions, *GSK-3β* expression was significantly suppressed (Fig. 6E). Immunofluorescence experiments further confirmed that overexpression of CDH11 enhances β-catenin protein expression levels while reducing GSK-3β protein expression levels; In contrast, co-transfection of CDH11 with miR-205a mimetic significantly attenuated these effects, though expression levels remained markedly higher than in the miR-205a mimetic-only group, with GSK-3β exhibiting the opposite trend (Fig. 6D, F).

## Discussion

This study suggests that thiram may involve a variety of biological effects in the process of inducing abnormal bone metabolism. Here, we further explore the relationship between the general toxicity of thiram and its specific effects on key signaling pathways, aiming to provide a more comprehensive understanding of its mechanism in the disease model. As a dithiocarbamate pesticide, thiram is known to trigger a wide range of cellular stress responses, especially oxidative stress and mitochondrial dysfunction [26–28]. However, in a specific physiological or pathological context, thiram may also play a role through relatively specific molecular pathways. For example, this study observed that thiram interfered with key signaling molecules related to cartilage development, and these changes could not be completely explained by oxidative stress. Similarly, recent studies have suggested that thiram can affect cell differentiation by regulating β-catenin/GSK-3β, OPG/RANKL, and other signal axes closely related to bone metabolism [29–31], suggesting that it may have certain pathway selectivity under certain conditions. Therefore, in the model of abnormal bone metabolism induced by thiram, basic toxicity such as oxidative damage and energy crisis can extensively destroy cell homeostasis and indirectly affect the expression of cartilage-related genes; On the other hand, thiram may directly interfere with specific signal transduction or transcriptional regulatory networks, thus relatively specifically disrupting the process of bone remodeling.

During embryonic development, mesenchymal cells undergo aggregation and differentiate into chondrocytes, which subsequently secrete extracellular matrix (ECM) components including type II collagen and proteoglycans. This forms the cartilaginous template for future bone, which progressively expands through chondrocyte proliferation [32]. Prior to the initiation of endochondral ossification, chondrocytes in the predetermined ossification focus undergo hypertrophy. Newly differentiated osteoblasts deposit around the ossification focus to form a periosteal bone collar. Subsequently, blood vessels, osteoclasts, bone marrow, and osteoblast precursor cells invade the cartilage matrix from the bone collar, collectively establishing the primary ossification center [33, 34]. The center gradually expands toward both ends of the cartilage model, accompanied by osteoclast resorption of the cartilage ECM and osteoblast bone deposition on the residual cartilage scaffold. In long bones, secondary ossification centers subsequently form at both ends of the cartilage model, with the retained cartilage structure between them constituting the growth plate, while the bone ends remain as permanent articular cartilage [35, 36]. The growth plate, as the key structure for longitudinal bone growth during endochondral ossification, coordinates skeletal development and maturation by strictly regulating the proliferation, maturation, and apoptosis of chondrocytes. In this process, chondrocytes undergo hypertrophy, matrix mineralization, apoptosis, vascular invasion, and osteoblast recruitment, ultimately achieving the replacement of cartilage with bone [37]. However, TD disrupts this process, primarily manifested by abnormal angiogenesis in the growth plate region and reduced vascular infiltration in the hypertrophic zone. This leads to inadequate nutrient supply for osteoblasts, osteoclasts, and mesenchymal cells, thereby impeding cartilage calcification. Simultaneously, extensive chondrocyte injury disrupts proliferation, differentiation, and apoptosis, along with abnormal ECM metabolism, thereby impeding subsequent bone deposition [38, 39]. Previous studies have demonstrated significant dysregulation in the expression of multiple cartilage matrix proteins and key skeletal development factors (such as Runx2, COL2A1, BMP2, MMP13, COL10A1, ACAN, Sox9, OPN, and TGF-β) in TD tibiae [40–42]. Particularly in the TD model, Runx2 and BMP2 expression is downregulated, core ECM components like ACAN and type II collagen are reduced, and the expression of Sox9, a key regulator of chondrocyte differentiation, is significantly diminished in the growth plate [43, 44]. These findings indicate that the pathogenesis of TD is closely associated with the suppression of multiple chondrogenic genes. The downregulation of chondrogenic differentiation-related genes observed in this study further supports this notion, highlighting the central role of developmental differentiation gene dysfunction in the occurrence of TD.

MicroRNAs have become effective regulators of cell gene expression. They participate in post-transcriptional regulation of almost all cell processes, and therefore play an important role in the development of many diseases. Therefore, miRNA as a potential new tool for diagnosis and even treatment has aroused great interest. miRNAs mainly regulate geneexpression by targeting mRNAs for degradation and/or translational repression. Therefore, changes in miRNA expression levels will affect the process of target regulation, thereby affecting cellular homeostasis [45, 46]. miR-205, as the only microRNA expressed when myocardial cells stop growing, has been shown to increase heart mass by 35% in animals with myocardial cell-specific miR-205 deficiency, confirming the potential role of miR-205 in controlling myocardial cell proliferation [47]. The expression of miR-205 is time-dependent during bone induction, and miR-205 can regulate the protein expression of runt-related transcription factor 2 (Runx2) and reverse the negative effects of miR-205 on osteoblast differentiation [48]. Therefore, miR-205 is believed to play a role in bone development. Wu et al. [49] further identified miR-205a as a growth-related gene in chickens. In this study, the up-regulation of miR-205a level in chondrocytes treated with thiram, providing evidence that broiler chickens develop tibial chondrodysplasia with an up-regulation in miR-205a expression. At the same time, we found that overexpression of miR-205a downregulated the expression of cartilage development related genes such as Runx2, while inhibition of miR-205a upregulated the expression of cartilage development related genes such as Runx2, demonstrating that miR-205a can regulate the development and growth of tibial cartilage. By inhibiting the expression of miR-205a, we reversed the effect of thiram on tibial cartilage dysplasia in broiler chickens. CDH11 is a key regulatory factor for bone growth in animal organisms, with the function of maintaining bone mass and differentiating into osteoblasts [50]. In our study, the level of CDH11 did indeed down-regulated in chondrocytes poisoned with thiram, confirming that miR-205a expression was up-regulated in broiler tibial chondrodysplasia and concurrently inhibited the level of CDH11. We validated the mutual targeting relationship between miR-205a and CDH11 through dual luciferase assay. Therefore, we constructed an overexpression vector for CDH11 and found that genes related to cartilage development, such as Runx2, were upregulated, indicating that CDH11 may regulate tibial cartilage development through signaling pathways.

Li et al. [51, 52] reported that the up-regulation of CDH11 can reverse the inhibitory of Wnt/β-catenin pathway by extracellular vesicles, which aligns with the experimental evidence presented in this paper demonstrating that CDH11 overexpression can stimulate the Wnt/β-catenin pathway. Our experiments have found that the Wnt pathway is an indispensable part of chondrocyte proliferation, migration, and differentiation processes [53]. The Wnt signaling pathway is evolutionarily conserved and stable, and the Wnt/β-catenin signaling pathway is one of the important pathways regulating cell osteogenic differentiation. Its main components include Wnt protein, β-catenin, Frizzled receptor, and protein glycogen synthesis kinase 3β (GSK-3β), which participate in regulating biological processes such as cell proliferation, differentiation, and embryonic development. When the Wnt/β-catenin pathway is activated, stem cells tend to differentiate into osteoblasts, while inhibition of the pathway promotes chondrogenic differentiation [54]. In our previous in vivo study using a broiler chicken model of thiram-induced TD, we demonstrated that baicalin treatment significantly alleviated TD pathology. Mechanistically, this therapeutic effect was associated with the modulation of miR-205a and CDH11 expression, as well as the regulation of Wnt/β-catenin pathway activity in cartilage tissue [55]. These in vivo observations provide correlative context for our current in vitro findings, as they align with the proposed involvement of the miR-205a–CDH11 axis in regulating Wnt/β-catenin signaling during chondrocyte differentiation. In our experiment, thiram treatment resulted in down-regulation of Wnt/β-catenin-related genes in chondrocytes, confirming that chondrodysplasia in broiler chickens inhibits this pathway, consequently impairing chondrocyte differentiation into osteoblasts. During thiram-induced tibial dysplasia, inhibition of the Wnt pathway can promote the expression of GSK-3β. Lithium chloride acts as a β-catenin agonist by inhibiting GSK-3, resulting in β-catenin accumulation and the inhibition of chondrogenic differentiation [56]. Upon treatment with the lithium chloride pathway activator in our experiment, the pathway was activated, leading to up-regulation of cartilage-related gene expression and facilitating cartilage development. It has been verified that the development of tibial cartilage is closely related to the Wnt/β-catenin signaling pathway, which is a key factor in bone development balance and plays an important role in regulating chondrocyte proliferation and bone tissue stability.

In conclusion, miR-205a acts as a crucial regulator in the progression of chondrodysplasia by directly inhibiting the expression of CDH11. This inhibition of CDH11, in turn, inhibits the expression of the Wnt pathway, leading to down-regulation of cartilage-related gene expression and the development of tibial cartilage dysplasia. The results of this study provide new insights into the pathogenesis of chondrodysplasia and confirm that miR-205a could serve as a potential biomarker for diagnosing this condition. Furthermore, this study identifies potential drug treatments for chondrodysplasia.

In the discussion part of this study, we recognize that there are some limitations in the current work, which need to be further deepened and improved in the future. First, at the mechanism level, due to the number of molecular indicators detected, there may be omissions of other key regulatory links in the pathway, resulting in incomplete description of the upstream and downstream relationship network; At the same time, GSK-3β is in a complex intracellular regulatory network, and its activity changes may also have other explanations not involved in this study. In addition, in addition to the verified targets, miR-205a may also regulate other genes not included in the analysis, and these target genes may also participate in the regulation of related biological processes. Therefore, the current results do not fully reflect its overall regulatory network. In terms of experimental methods, the simultaneous overexpression of CDH11 and miR-205a using two independent vectors may cause competitive transfection effect, potentially reducing their respective expression efficiency, which may be one of the reasons for the insignificant difference in CDH11 expression in some experiments. Future studies may consider using a single vector co-expression strategy to ensure a more consistent and balanced gene expression level. Most importantly, it must be emphasized that although the Thiram-induced model is a well-established tool for studying dyschondroplasia, it may involve intrinsic toxic effects that are not exclusively attributable to the miR-205a–CDH11–Wnt/β-catenin axis. As such, the molecular and phenotypic changes observed in this model should not be interpreted as purely or specifically attributable to the miR-205a–CDH11–Wnt/β-catenin axis alone. This constitutes a critical limitation, as it precludes a definitive distinction between pathway-specific mechanisms and broader toxicological influences. Therefore, the current evidence remains preliminary, and more rigorous experimental designs are necessary to establish causal relationships and to fully elucidate the underlying mechanisms.

## Materials and methods

### Cell culture

This study employed chicken embryo tibial chondrocytes, leveraging the research group’s established technical platform [57]. The chicken embryo serves as a classical model due to its transparent cartilage structure, which offers clear visualization and ease of manipulation. The selection of the thiram induced chondrodysplasia model continues the group’s previously validated approach, which efficiently simulates chondrocyte apoptosis and matrix abnormalities.

Take 20 tibial cartilages from 15-day-old chicken embryos and cut into smaller pieces using scissors. Afterwards, the clipped cartilage was moved into a petri dish. Next, a 0.1 mg/mL type IV collagenase solution (dissolved in PBS) was prepared and heated, and it was then added to the petri dish. The dish was placed in the cell culture incubator at 37 °C for overnight digestion. The cells were suspended in 6 mL of 15% medium (serum+double antibodies+DMEM/F12 medium). Cell counts were performed, and then placed in a 37 °C, 5% CO_2_ balanced humidity incubator for incubation. Following digestion and filtration, an average yield of approximately (2.0 ± 0.2) × 10^7^ viable tibial chondrocytes was obtained per isolation. Cells were seeded at appropriate densities depending on the plate format, as specified in each assay. Of note, primary chondrocytes exhibit delayed adherence (requiring approximately 12 h for complete attachment) and a slow proliferation rate. Consequently, higher seeding densities than those typically used for cell lines were required for downstream assays. For a 24-well plate (with 500 μL cell dilution added to each well), chondrocytes were seeded at a density of 5 × 10⁵ cells/mL. This relatively high density was used to achieve optimal confluence (80 ~ 90%) at the time of transfection, which minimizes reagent cytotoxicity while ensuring sufficient cell yield for downstream analyses. For a 12-well plate (with 1000 μL cell dilution added to each well), the seeding density was 1 × 10⁶ cells/mL, and for a 96-well plate (with 100 μL cell dilution added to each well), it was 1 × 10^5^ cells/mL.

### Analysis of cell viability and proliferation

Primary chondrocytes were isolated from avian embryonic growth plates. A model of differentiation impairment was established by treating these cells with thiram (5 μM), as previously described in our studies [40] (Wu et al., 42). This regimen recapitulates stable dyschondroplasia phenotypes without significant cytotoxicity. Through repeated experiments, it was found that treating chondrocytes with 5 μM thiram for 24 h induced changes in chondrogenic differentiation-related genes consistent with the TD model while not causing significant cell mortality. Therefore, a concentration of 5 μM was selected for subsequent experiments. Briefly, Chondrocytes were seeded in 96-well plates at a density of 1 × 10^5^ cells/mL. After treatment with 5 μM thiram for 24 hours, cell viability and proliferation were assessed using a Cell Counting Kit-8 (C6005, NCM) with an incubation period of 1 to 2 h.

Chondrocytes were seeded in 24-well plates at a density of 5 × 10^5^ cells per mL and transfected separately with miR-205a mimics, miR-205a inhibitor, or a non-targeting control (NC; final concentration 75 nM) using Lipofectamine^TM^ 3000 transfection reagent, according to the manufacturer’s instructions. Briefly, each oligonucleotide was first diluted with DEPC water to prepare a 20 μM stock solution. For transfection in a 24-well format, 0.75 μL of Lipofectamine^TM^ 3000 reagent and 1 μL of P3000^TM^ reagent were used per well to form transfection complexes. After 24 h of transfection, the medium was replaced with fresh complete medium at 24 h, and cells were harvested for subsequent viability and proliferation assays. For other plate formats, reagent volumes were scaled proportionally based on the surface area.

Chondrocytes were seeded in 96-well plates at a density of 1 × 10^5^ cells per mL and were treated with thiram (5 μM) for 24 h and then transfected with both miR-205a inhibitor and miR-205a mimics. Cells were collected after 24 h for cell activity and cell proliferation assays.

### Toluidine blue staining

Toluidine blue staining, as a commonly used cell staining method, is often used to identify sulfated glycosaminoglycans in chondrocytes. Firstly, the cell culture dish was removed from the incubator and washed with PBS at room temperature, after which subsequent experimental operations were carried out according to the manufacturer’s instructions (G1032-100ML, Servicebio, China). In this study, toluidine blue staining was used to qualitatively identify chondrocytes, and cell phenotype was confirmed by observing cell morphology and metachromatic phenomenon, without quantitative analysis.

### Dual-luciferase reporter assay

The potential targeting binding sites of miR-205a on CDH11 were predicted using the TargetScan bioinformatics software. A recombinant reporter plasmid was constructed by inserting the from *Gallus gallus* CDH11-3’UTR sequence downstream of the luciferase gene in an empty pGL3 vector. The constructed plasmid was co-transfected into HEK-293T cells. At 48 hours post-transfection, the cells were harvested and lysed. The dual-luciferase reporter assay was then performed strictly according to the manufacturer’s instructions for the assay kit (catalog No. RG027, Beyotime, China). Renilla luciferase was used as an internal control, and the firefly luciferase activity was normalized to the Renilla luciferase activity to account for variations in transfection efficiency.

### Reverse transcription quantitative polymerase chain reaction (RT-qPCR)

The culture plate was washed 1 time with PBS and added 500 μL TRIzol RNA (AG21102, AG). Following the manufacturer’s instructions, the total isolated RNA was quantified. A cDNA synthesis kit was employed to generate single-stranded cDNA from the RNA, and the synthesized cDNA was subsequently stored at −80 °C. Quantitative real-time PCR (qPCR) was performed using a PCR reagent kit (R22301, Vazyme) with the SYBR Green master mix method on a LightCycler 480 real-time PCR instrument (Roche, USA). The thermal cycling protocol was set as follows: initial pre-denaturation at 95 °C for 30 s; followed by 40 cycles of denaturation at 95 °C for 5 s and annealing/extension at 60 °C for 30 s. A melting curve analysis was subsequently performed to verify the specificity of the amplification [58]. Each sample was assayed in triplicate (three technical replicates), and the entire experiment was independently repeated four times (*n* = 4). The relative expression levels of the target genes were calculated using the 2^(-ΔΔCt) method, with GAPDH serving as the internal reference gene for normalization. All primer sequences used in this study were designed based on the Gallus genome; detailed information is provided in Table S1.

### Western blotting analysis

The culture plate was washed two times with PBS. Then, RIPA lysis buffer (P0013K, Beyotime, China) along with Phenylmethanesulfonyl fluoride buffer were added to the culture plate in a ratio of 100:1. Protein concentration was determined using a BCA protein assay kit (WB6501, NCM Biotech). The lysates were diluted with RIPA + PMSF solution to a final concentration of 1.25 μg/μL. Loading buffer was then added at a ratio of 4:1 (v/v, protein solution: loading buffer), and the mixture was boiled for 10 min, followed by centrifugation at 12,000 rpm for 5 min. For each lane, 8 μL of the prepared sample (containing 10 μg of total protein) was loaded onto an 12% SDS-polyacrylamide gel. Proteins were separated by electrophoresis and subsequently transferred onto a polyvinylidene difluoride (PVDF) membrane (0.45 µm pore size) using electroblotting. The membrane was blocked with 5% non-fat milk in TBST for 1 h at room temperature, and then incubate with the primary antibody diluted to the appropriate proportion with antibody diluent (WB100D, NCM biotech) at 4 °C for 14 ~ 16 h. Following that, membranes were incubated with horseradish peroxidase (HRP)-conjugated goat anti-rabbit or anti-mouse secondary antibodies (CW0103/CW01025, CWBIO) diluted 1:5000 in TBST, at room temperature for 1 h. A developer solution was prepared, and the gel was photographed [59]. Information regarding the antibodies utilized in this study can be found in Table S2.

### Immunostaining

The plates were fixed using 4% paraformaldehyde heated to 37 °C for 20 min. Then, the plates were treated with 0.3% Triton-X-100 for 20 min. Select the target antibody and incubate overnight, and then dilute the goat anti rabbit and goat anti mouse antibodies in proportion and incubate in the dark for 1 h. Use anti fluorescence quenching sealing agent containing DAPI (P0131, Beyotime, China) for sealing [60].

### Statistical analysis

Data are presented as mean ± SD from at least three independent experiments. Data were analyzed using GraphPad Prism 9 (San Diego, California, USA). For analyzing differences between two groups, t-tests were employed to determine statistical significance. For three or more groups, one-way ANOVA was conducted, followed by Dunnett’s t-tests for multiple comparisons. Horizontal lines in figures indicate comparisons between the means of two groups. Statistical significance was set at ****p < 0.0001, ***p < 0.001, **p < 0.01, and *p < 0.001.

## Supplementary information

Original Data: Uncropped western blots

SUPPLEMENTAL MATERIAL_Tables

## Data Availability

The datasets used and analyzed during the current study are available from the corresponding author on reasonable request.
