# Supplementary material for: miR-205a mediated suppression of CDH11 disrupts Wnt/β-catenin signaling and impairs chondrocyte differentiation
Source: Cell Death Discov. 2026 May 6;12:284. doi: 10.1038/s41420-026-03146-3 (PMC13315870; doi:10.1038/s41420-026-03146-3)
Supplement: Supplementary file 1 — Original Data: Uncropped western blots [file 41420_2026_3146_MOESM1_ESM.pdf]

miR-205a Mediated Suppression of CDH11 Disrupts Wnt/β-Catenin Signaling and Impairs Chondrocyte Differentiation

Kai Liu[1], Buyun Chen[1], Junhong Hou[1], Yuanliang Li[1], Lihong Ning[2], Shaochuan Li[1], Ying Li[1], Aoyun Li[3*], Quazi T. H. Shubhra[4*], Hui Zhang[1,5*]

[1]*College of Veterinary Medicine, South China Agricultural University, Guangzhou 510642, China.*

[2]*Xizang Animal Disease Prevention and Control Center, Lasa 85v0032, China.*

[3]*College of Veterinary Medicine, Henan Agricultural University, Zhengzhou 450046, China.*

[4]*Institute of Chemistry, University of Silesia in Katowice, Szkolna 9, 40-006 Katowice, Poland.*

[5]*College of Animal Science, Xizang Agriculture and Animal Husbandry, Linzhi 860000, China.*

[*]Corresponding author: Hui Zhang; E-mail: hz236@scau.edu.cn

College of Veterinary Medicine, South China Agricultural University, Guangzhou 510642, China.

College of Animal Science, Xizang Agriculture and Animal Husbandry, Linzhi 860000, China.

[*]Corresponding author: Quazi T. H. Shubhra; E-mail: tanminul-haque-shubra.quazi@us.edu.pl

Institute of Chemistry, University of Silesia in Katowice, Szkolna 9, 40-006 Katowice, Poland.

[*]Corresponding author: Aoyun Li; E-mail: aoyunli@sina.cn

College of Veterinary Medicine, Henan Agricultural University, Zhengzhou 450046, China.

Supplementary Figure

Uncropped gels and blots for Figures 1–5

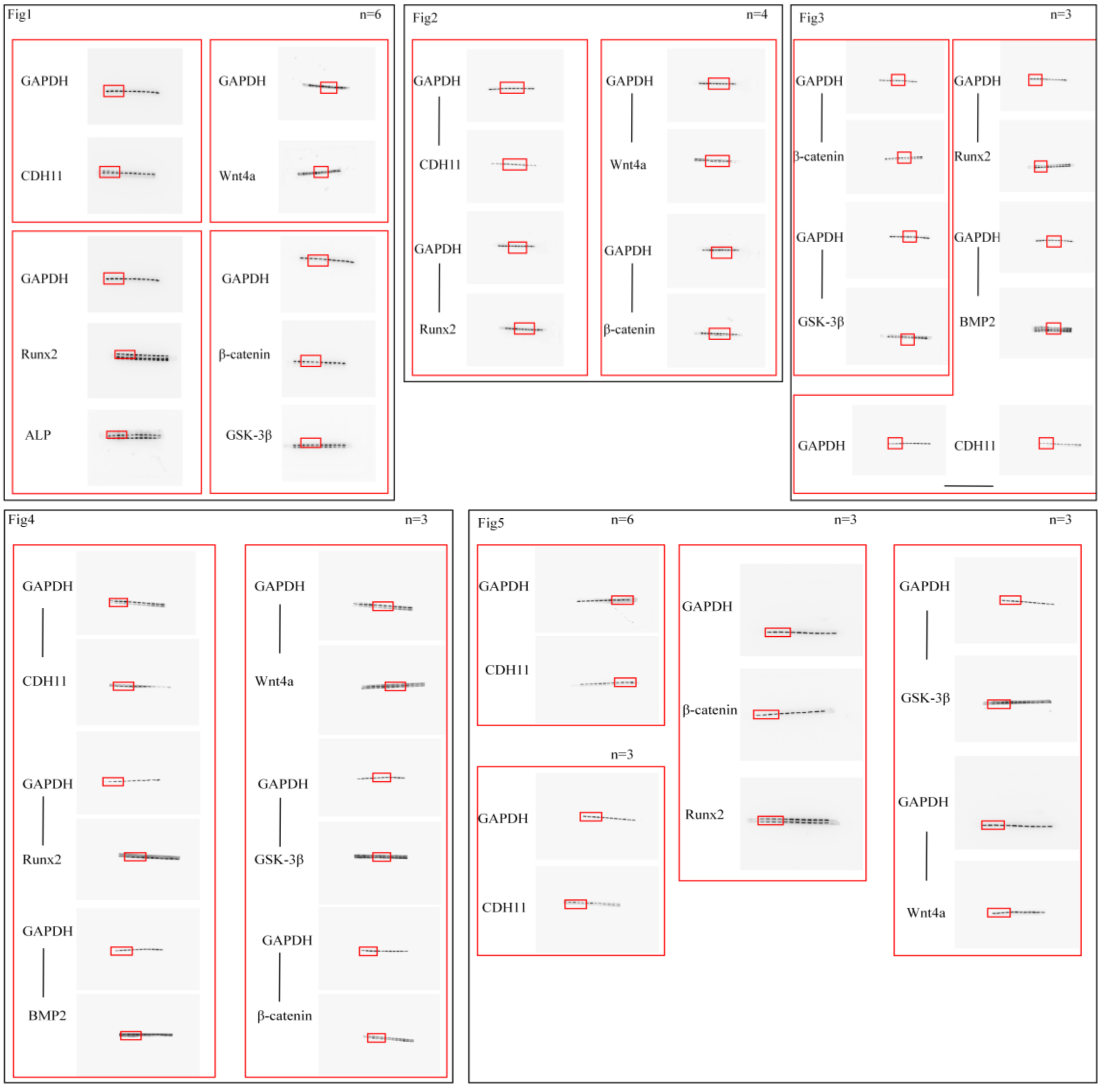

Description: Uncropped western blot images corresponding to the main figures are shown. Red boxes indicate the regions displayed in the main figures. During Western blotting, gels were physically cut prior to membrane transfer to allow parallel antibody incubation for different target proteins. Each image represents the full scanned membrane for the indicated protein, with no additional digital cropping or editing.
