# Supplementary material for: miR-205a mediated suppression of CDH11 disrupts Wnt/β-catenin signaling and impairs chondrocyte differentiation
Source: Cell Death Discov. 2026 May 6;12:284. doi: 10.1038/s41420-026-03146-3 (PMC13315870; doi:10.1038/s41420-026-03146-3)
Supplement: Supplementary file 2 — SUPPLEMENTAL MATERIAL_Tables [file 41420_2026_3146_MOESM2_ESM.pdf]

miR-205a Mediated Suppression of CDH11 Disrupts Wnt/β-Catenin Signaling and Impairs Chondrocyte Differentiation

Kai Liu[1], Buyun Chen[1], Junhong Hou[1], Yuanliang Li[1], Lihong Ning[2], Shaochuan Li[1], Ying Li[1], Aoyun Li[3*], Quazi T. H. Shubhra[4*], Hui Zhang[1,5*]

[1]*College of Veterinary Medicine, South China Agricultural University, Guangzhou 510642, China.*

[2]*Xizang Animal Disease Prevention and Control Center, Lasa 85v0032, China.*

[3]*College of Veterinary Medicine, Henan Agricultural University, Zhengzhou 450046, China.*

[4]*Institute of Chemistry, University of Silesia in Katowice, Szkolna 9, 40-006 Katowice, Poland.*

[5]*College of Animal Science, Xizang Agriculture and Animal Husbandry, Linzhi 860000, China.*

*Corresponding author: Hui Zhang; E-mail: hz236@scau.edu.cn

College of Veterinary Medicine, South China Agricultural University, Guangzhou 510642, China.

College of Animal Science, Xizang Agriculture and Animal Husbandry, Linzhi 860000, China.

*Corresponding author: Quazi T. H. Shubhra; E-mail: tanminul-haque-shubra.quazi@us.edu.pl

Institute of Chemistry, University of Silesia in Katowice, Szkolna 9, 40-006 Katowice, Poland.

*Corresponding author: Aoyun Li; E-mail: aoyunli@sina.cn

College of Veterinary Medicine, Henan Agricultural University, Zhengzhou 450046, China.

Table S1. Primer sequence information

Genes	Primer sequence (5'-3')	Accession No.	genus
CDH11	(F) AACAGACCTTTGGAACCGCC<br>(R) CGGCACATTGGCATGGTAGT	NM_001004371.1	Gallus
RUNX2	(F) CCCTGAACGCCTCAGTGATT<br>(R) GCTCAAGTAGGACGGGTACG	NC_052534.1	Gallus
COL2a1	(F) GCCACCCTCAAATCCCTCAA<br>(R) ACTCGGGATGGCAGAGTTTG	NC_052565.1	Gallus
MMP13	(F) TTTGGGCTATGAATGGCTAT<br>(R) TAGTATGCAGGATGCGGACA	NC_052532.1	Gallus
BMP2	(F) TCAGCTCAGGCCGTTGTTAG<br>(R) GTCATTCCACCCCACGTCAT	NC_052534.1	Gallus
Wnt4a	(F) GGAGTGCCAGTACCAATTCC<br>(R) CGTCGAATTTCTCCTTCAGC	NC_052552.1	Gallus
β-catenin	(F) CCTGGTGCTGACTACCCAGT<br>(R) CTCAGCAACTCTACAGGCCAA	NC_052533.1	Gallus
GSK-3β	(F) ACTGCAGTCCTATGGAGTTGA<br>(R) ATATGCCACATCCTCTGCACAT	NC_052532.1	Gallus
PKC	(F) CAGATTGTTGGATGGCACAC	NC_052549.1	Gallus

	(R) ATGCTGACGAGGATGGAAAT		
GAPDH	(F) AGTCAACGGATTTGGCCGTA<br>(R) TTCCCGTTCTCAGCCTTGAC	NC_052532.1	Gallus

Table S2. List of antibodies used during the study.

Target protein	Company	Category	Product number	Source species	Dilution ratio	Molecular size
CDH11	ABClonal	Polyclonal	A8110	Rabbit	1：1000	110 kDa
RUNX2	ABClonal	Polyclonal	A11753	Rabbit	1：1000	60 kDa
COL2a1	ABClonal	Polyclonal	A28341	Rabbit	1：2000	141 kDa
BMP2	ABClonal	Polyclonal	A27101	Rabbit	1：3000	15 kDa
ALP	ABClonal	Polyclonal	A6346	Rabbit	1：5000	25-35 kDa
Wnt4	ABClonal	Polyclonal	A7809	Rabbit	1：1000	46 kDa
β-catenin	ABClonal	Polyclonal	A19657	Rabbit	1：10000	100 kDa
GSK-3β	ABClonal	Polyclonal	A27666	Rabbit	1：10000	46 kDa
